# Predictors of epicardial adipose tissue in patients with type 2 diabetes mellitus

**DOI:** 10.1186/1758-5996-6-55

**Published:** 2014-05-09

**Authors:** Emin M Akbas, Hikmet Hamur, Levent Demirtas, Eftal M Bakirci, Adalet Ozcicek, Fatih Ozcicek, Ufuk Kuyrukluyildiz, Kultigin Turkmen

**Affiliations:** 1 Department of Endocrinology, Erzincan University Mengucek Gazi Training and Research Hospital, Erzincan, Turkey; 2 Department of Cardiology, Erzincan University Mengucek Gazi Training and Research Hospital, Erzincan, Turkey; 3 Department of Internal Medicine, Erzincan University Mengucek Gazi Training and Research Hospital, Erzincan, Turkey; 4 Department of Anesthesiology and Reanimation, Erzincan University Mengucek Gazi Training and Research Hospital, Erzincan, Turkey; 5 Department of Nephrology, Erzincan University Mengucek Gazi Training and Research Hospital, Erzincan, Turkey

**Keywords:** Diabetes mellitus, Waist circumference, Platelet-to-lymphocyte ratio, Neutrophil-to-lymphocyte ratio, Epicardial adipose tissue, Atherogenic index of plasma

## Abstract

**Background:**

Epicardial adipose tissue (EAT), visceral fat depot of the heart, was found to be associated with coronary artery disease in cardiac and non-cardiac patients. Platelet-to-lymphocyte ratio (PLR) and neutrophil-to-lymphocyte ratio (NLR) were introduced as potential markers to determine inflammation in various disorders. Recently, atherogenic index of plasma (AIP) was found to be closely associated with atherosclerosis in general population. Waist circumference is commonly used to assess the risk factors in various metabolic disorders. There has been a well known relation between inflammation and peripheral adipose tissue in diabetes mellitus. However, the data regarding EAT and inflammation is scant in this population. Hence, we aimed to determine the relationship between PLR, NLR, AIP, waist circumference and EAT in diabetic patients.

**Methods:**

This was a cross-sectional study involving 156 patients with type 2 diabetes mellitus (87 females, 69 males; mean age, 53.62 ± 9.33 years) and 50 control subjects (35 females, 15 males; mean age, 51.06 ± 8.74 years). EAT was measured by using a trans-thoracic echocardiogram. Atherogenic index of plasma was calculated as the logarithmically transformed ratio of the serum triglyceride to high density lipoprotein (HDL)cholesterol. NLR and PLR were calculated as the ratio of the neutrophils and platelets to lymphocytes, respectively.

**Results:**

Waist circumference, PLR, NLR, AIP and EAT measurements were significantly higher in diabetic patients when compared to control subjects. When diabetic patients were separated into two groups according to their median value of EAT (Group 1, EAT < 4.53 (n = 78) and group 2, EAT ≥4.53 (n = 78)), group 2 patients had significantly higher Body mass index (BMI), waist circumference, AIP, NLR and PLR levels. In the bivariate correlation analysis, EAT was positively correlated with PLR, NLR, AIP, BMI and waist circumference (r = 0.197, p = 0.014; r = 0.229, p = 0.004; r = 0.161, p = 0.044; r = 0.248, p = 0.002; r = 0.306, p < 0.001, respectively). Waist circumference was found to be independent variables of EAT.

**Conclusions:**

Simple calculation of PLR and measurement of waist circumference were found to be associated with increased EAT in diabetic patients.

## Background

Cardiovascular diseases remain the most common cause of morbidity and mortality in diabetic patients [1]. Nowadays, beside the main factors including hypertension, obesity, and dyslipidemia, novel risk factors such as chronic low-grade inflammation, advanced glycolisation end-products (AGE), oxidative stress and endothelial dysfunction are accepted as the responsible factors to highlight this increased cardiovascular risk in this population [2,3]. Epicardial adipose tissue (EAT), a metabolically active visceral fat tissue located between the heart and pericardium, has been proposed a novel cardiovascular risk in general population [4]. In the past years, high sensitive C-reactive protein (hs-CRP) was the most commonly encountered marker for evaluating inflammation. Recently, two novel inflammatory markers, neutrophils-to-lymphocyte ratio (NLR) and platelet-to-lymphocyte ratio (PLR), were demonstrated that these two markers could predict inflammation as accurate as hs-CRP in various disorders [5,6].

Anthropometric parameters are commonly used to assess the risk factors in various metabolic disorders [7]. Previous studies have shown that anthropometric parameters including waist circumference, body mass index, waist hip ratio, and waist height ratio are useful measures for predicting the incidence of type 2 diabetes mellitus in different patient groups [8,9]. Logarithmic ratio of triglycerides to HDL was defined as atherogenic index of plasma (AIP) and this index was found to be closely associated with atherosclerosis in general population [10]. However, in the literature, data regarding the relationship between anthropometric measures, PLR, NLR, AIP and EAT in diabetic patients is scant. Hence, we aimed to investigate the relationship between these parameters and EAT. We also sought to determine the independent variables of EAT in patients with type 2 diabetes mellitus and to compare these results with the others that obtained from control subjects.

## Methods

### Patients

The study protocol was approved by the Medical Ethics Committee of Erzincan University (School of Medicine, Erzincan, Turkey). Written informed consent was obtained from all subjects included in the study. This was a cross-sectional study involving 156 type 2 diabetic patients (87 females, 69 males; mean age, 53.62 ± 9.33 years; diabetes duration, 88.31 ± 75.35 months) and 50 control subjects (35 females, 15 males; mean age, 51.06 ± 8.74 years).

Patients aged 18–80 years willing to participate were screened. A review of medical records (including information on age, sex, weight, height, disease duration, medications, history of other diseases, smoking) was undertaken. Exclusion criteria were infection, autoimmune disease, and acute diabetic complications. One hundred and sixty patients were evaluated and 4 patients excluded from the study. Of these 4 patients, three patients had active infection; and one patient had diabetic ketoacidosis.

The remaining 156 type 2 diabetic patients fulfilled the above criteria and were enrolled in the study. Of these 156 patients, 29 patients were taking insulin and oral antidiabetics; 69 patients were taking only oral antidiabetics; 25 patients were taking only insulin and 33 patients were not taking any antidiabetic medication.

Control subjects were chosen from metabolically healthy individuals according to NCEP ATP III Metabolic syndrome criteria (Two or less metabolic criteria; Blood pressure ≥130/85 mmHg, TG ≥1.7 mmol/L, HDL-C: Men <1.03 mmol/L, Women <1.30 mmol/L, Glucose ≥5.6 mmol/L, Waist circumference: Men <102 cm, Women < 88 cm) [11].

### Biochemical analyses, data collection and procedures

The systolic blood pressure (SBP) and diastolic blood pressure (DBP) of patients were measured in the upright sitting position after >5 minutes of rest using an Erka sphygmomanometer (PMS Instruments Limited, Berkshire, UK) with an appropriate cuff size. Two readings were recorded for each individual. The mean value of two readings was defined as the blood pressure. Patients who were already on antihypertensive treatment (n = 70) or with SBP and DBP >140 mmHg and 90 mmHg (seventeen patients without taking any antihypertensive treatment) respectively were assumed to be hypertensive.

Body-mass index (BMI) was calculated as body weight divided by the square of the height. Waist circumference was measured at the level of midway between the lower rib margin and iliac crest after removal of the clothes. Weight and height was measured with the possible lightest clothing and without shoes. Estimated glomerular filtration rate (eGFR) was calculated according to Cockcroft-Gault formula [12].

Venous blood samples for biochemical analyses were drawn after at least 10 hours of fasting before taking any medication. All biochemical analyses were undertaken in the Central Biochemistry Laboratory of the Erzincan University School of Medicine, Mengücek Gazi Training and Research Hospital. Total cholesterol (TC), low density lipoprotein cholesterol (LDL-C), high density lipoprotein cholesterol (HDL-C) and plasma triglyceride concentrations were undertaken using an oxidize-based technique by the Beckman Coulter AU 2700 plus, Missima, Japan. Hemoglobin A1c (HbA1c) was measured by HPLC method using the Adams HA-8160 (Shiga, Japan). Serum creatinine were measured with Jaffe Method.

### Definition of PLR and NLR

Complete blood counts with automated differential counts, which included total white blood cells, neutrophils, platelets and lymphocytes, were obtained. NLR and PLR were calculated as the ratio of the neutrophils and platelets to lymphocytes respectively, both obtained from the same automated blood sample at the admission of the study.

### Definition of atherogenic index of plasma

Atherogenic index of plasma was calculated as the logarithmically transformed ratio of the serum triglyceride to HDL-cholesterol [13].

### Definition of epicardial adipose tissue thickness

All participants underwent transthoracic echocardiography imaging using an echocardiograph equipped with a broadband transducer (Vivid S4, GE Medical Systems, USA). Measurements were obtained from the long axis and apical four-chamber-view according to the standard criteria. Echocardiograms were recorded on videotapes. EAT appears as an echo-free space in the pericardial layers on 2-D echocardiography. EAT thickness was measured on the free wall of right ventricle at end-diastole from the parasternal long- and short-axis views by two cardiologists (E.M.B and H.H) blinded to clinical data. Measurements from the parasternal long- and short-axis were averaged.

The echocardiograms of 20 patients were randomly selected and a second measurement of the EAT was performed 2 weeks later in order to assess the inter-observer and intra-observer variability. The inter-observer and intra-observer variabilities of the EAT were found as 3.4% and 3.0%, respectively.

### Statistical analysis

Statistical analyses were carried out using the Statistical package for Social Sciences for Windows version 15.0 (SPSS, Chicago, IL, USA). Descriptive statistics for each variable were determined. Results for continuous variables were demonstrated as mean ± standard deviation. Statistical significant difference between the groups was determined by the chi-square test for categorical variables and unpaired Student *t* test for continuous variables. Associations between the variables were explored using the Pearson correlation and Spearman’s rho (for data that were not normally distributed). Logistic regression analysis with backward elimination was also performed to define variables associated with EAT. A *p* value less than 0.05 was considered significant.

## Results

### Baseline characteristics of patients

The baseline characteristics of 156 DM patients and 50 control subjects were shown in Table 1. There were no differences with respect to the following variables between diabetic patients and control subjects, age, gender, BMI, waist circumference, DBP, serum levels of triglyceride, TC, LDL-C and eGFR. Control group had significantly lower HbA1c, SBP, serum creatinine, AIP, EAT, NLR and PLR levels while HDL-C and uric acid levels were significantly higher in this group.

**Table 1:** Demographic, clinic and laboratory features of the study groups

**Parameters**	**Diabetic patients (n = 156)**	**Control group (n = 50)**	**P value**
Age (years)	53.62 ± 9.33	51.06 ± 8.74	0.080
Female/Male	87/69	35/15	0.980
BMI (kg/m^2^)	31.21 ± 5.87	32.86 ± 7.52	0.159
Waist circumference	103.24 ± 11.29	102.54 ± 11.28	0.702
HbA1c (%)	9.14 ± 2.45	5.50 ± 0.32	<0.001
Disease duration (months)	88.31 ± 75.35	-	-
SBP (mmHg)	135.80 ± 26.97	121.80 ± 16.41	0.001
DBP (mmHg)	74.49 ± 11.43	75.60 ± 11.50	0.553
Total cholesterol (mmol/L)	5.49 ± 1.36	5.61 ± 1.47	0.616
Triglyceride (mmol/L)	2.35 ± 1.55	1.97 ± 1.03	0.052
LDL - cholesterol (mmol/L)	3.39 ± 1.12	3.66 ± 0.85	0.078
HDL - cholesterol (mmol/L)	1.1 ± 0.23	1.23 ± 0.22	<0.001
AIP	0.27 ± 0.30	0.16 ± 0.26	0.012
eGFR (mL/min)	116.76 ± 40.81	126.07 ± 42.42	0.177
Creatinin (mg/dL)	0.82 ± 0.36	0.73 ± 0.16	0.017
EAT (mm)	4.66 ± 1.59	3.91 ± 1.60	0.005
NLR	1.90 ± 0.82	1.45 ± 0.38	<0.001
PLR	116.67 ± 41.62	102.97 ± 35.97	0.027
White blood cell (10^3^/μL)	7.42 ± 2.06	6.77 ± 1.58	0.022
Lymphocyte (10^3^/μL)	2.43 ± 0.73	2.55 ± 0.71	0.296
Neutrophil (10^3^/μL)	4.36 ± 1.59	3.57 ± 0.95	0.001
Platelet (10^3^/mm^3^)	265.87 ± 67.82	247.52 ± 57.76	0.087
Uric acid (mg/dL)	4.68 ± 1.50	5.30 ± 1.44	0.010

AIP; Atherogenic index of plasma, BMI; body mass index, EAT; epicardial adipose tissue NLR; neutrophils-to-lymphocyte ratio, PLR; Platelet-to-lymphocyte ratio.

### Evaluation of PLR, NLR, AIP and EAT

Platelet-to-lymphocyte ratio, NLR, white blood count (WBC) and neutrophil levels were significantly higher in diabetic patients when compared to control group (Table 1). Absolute lymphocyte and platelet counts had no significant difference (p > 0.05). When diabetic patients were separated into two groups according to their median value of EAT (Group 1, EAT < 4.53 (n = 78) and group 2, EAT ≥4.53 (n = 78)), there were no differences with respect to the following variables between these two groups; age, gender, HbA1c, disease duration, SBP, DBP, serum levels of triglyceride, TC, LDL-C, creatinine, uric acid and eGFR (Table 2). Group 2 patients had significantly higher BMI, waist circumference, AIP, NLR and PLR levels while HDL-C levels were significantly lower in this group (Table 2).

**Table 2:** Demographic, clinic and laboratory features of diabetic patients according to EAT groups

**Parameters**	**EAT < 4.53 (n = 78)**	**EAT ≥ 4.53 (n = 78)**	**P value**
Age (years)	52.80 ± 9.39	54.44 ± 9.27	0.273
Female/Male	46/32	41/37	0.519
BMI (kg/m^2^)	30.18 ± 6.00	32.24 ± 5.59	0.028
Waist circumference	100.30 ± 11.36	106.19 ± 10.49	0.001
HbA1c (%)	9.12 ± 2.55	9.16 ± 2.35	0.922
Disease duration (months)	82.62 ± 72.75	94.00 ± 77.92	0.347
SBP (mmHg)	135.77 ± 26.57	135.83 ± 27.54	0.988
DBP (mmHg)	73.78 ± 9.58	75.19 ± 13.05	0.443
Total cholesterol (mmol/L)	5.46 ± 1.37	5.53 ± 1.35	0.731
Triglyceride (mmol/L)	2.12 ± 1.18	2.57 ± 1.83	0.068
LDL - cholesterol (mmol/L)	3.46 ± 1.15	3.33 ± 1.09	0.468
HDL - cholesterol (mmol/L)	1.16 ± 0.22	1.04 ± 0.24	0.002
AIP	0.21 ± 0.28	0.33 ± 0.30	0.010
eGFR (mL/min)	115.51 ± 38.70	118.00 ± 43.04	0.705
Creatinin (mg/dL)	0.78 ± 0.25	0.86 ± 0.45	0.145
EAT (mm)	3.52 ± 0.75	5.80 ± 1.37	<0.001
NLR	1.73 ± 0.69	2.08 ± 0.90	0.007
PLR	109.14 ± 38.06	124.20 ± 43.85	0.023
Uric Acid (mg/dL)	4.62 ± 1.42	4.74 ± 1.58	0.628

AIP; Atherogenic index of plasma, BMI; body mass index, EAT; epicardial adipose tissue NLR; neutrophils-to-lymphocyte ratio, PLR; Platelet-to-lymphocyte ratio.

### Correlation analysis

In the bivariate correlation analysis in diabetic patients, EAT was positively correlated with PLR, NLR, AIP, BMI and waist circumference (Table 3). Atherogenic index of plasma was positively correlated with HbA1c and waist circumference (r = 0.197, P = 0.014 and r = 0.159, P = 0.048 respectively).

**Table 3:** Bivariate correlation results between EAT and other significant parameters in diabetic patients

**Parameters**	**r**_**S**_	**P value**
NLR	0.229	0.004
PLR	0.197	0.014
AIP	0.161	0.044
BMI (kg/m^2^)	0.248	0.002
Waist circumference	0.306	<0.001

AIP; Atherogenic index of plasma, BMI; body mass index, EAT; epicardial adipose tissue NLR; neutrophils-to-lymphocyte ratio, PLR; Platelet-to-lymphocyte ratio.

We also performed logistic regression analysis to define the variables of EAT (Table 4). Age, duration of diabetes, age, waist circumference, BMI, systolic blood pressure, hemoglobin A1C, uric acid, LDL-cholesterol, AIP, NLR and PLR were included in this model. Waist circumference was found to be independent variables of EAT.

**Table 4:** Variables of epicardial adipose tissue

**Parameters**	**Standardized beta**^**‡**^	**t**	**P value**	**95% CI**
STEP 1				
Age (years)	0.083	0.93	0.35	−0.016-0.44
Duration of diabetes (months)	0.115	1.35	0.17	−0.001-0.006
BMI (kg/m^2^)	−0.110	−0.78	0.43	−0.105-0.046
Waist circumference (cm)	0.382	2.159	0.01	0.013-0.095
SBP (mmHg)	−0.114	−1.324	0.18	−0.017-0.003
HbA1c (%)	−0.032	−0.368	0.71	−0.132-0.091
Uric acid	−0.012	−0.143	0.88	−0.193-0.167
LDL (mmol/L)	0.043	0.539	0.59	−0.004-0.007
AIP	0.093	1.106	0.27	−0.396-1.404
NLR	0.022	0.233	0.81	−0.315-0.399
PLR	0.117	1.287	0.20	−0.002-0.011
r^2^ = 0. 16				
Adjusted r^2^ = 0.096				
p = 0.007				
STEP 10				
PLR	0.141	1.867	0.06	0.000-0.011
Waist circumference (cm)	0.326	4.320	<0.0001	0.025-0.067
r^2^ = 0. 127				
Adjusted r^2^ = 0.116				
p < 0.0001				

AIP; Atherogenic index of plasma, BMI; body mass index, CI; confidental interval, EAT; epicardial adipose tissue NLR; neutrophils-to-lymphocyte ratio, PLR; Platelet-to-lymphocyte ratio.

## Discussion

There were five main findings of the present study. First, inflammation markers including PLR and NLR were significantly increased in diabetic patients when compared with control group. Second, AIP and EAT measurements were found to be increased in diabetic patients compared to control subjects. Third, AIP, NLR and PLR were significantly high in patients with higher EAT. Forth, EAT was positively correlated with PLR, NLR, BMI, waist circumference and AIP. Lastly, only waist circumference was found to be independent predictors of increased EAT in diabetic patients.

Chronic low-grade inflammation plays an important role in increased cardiovascular morbidity and mortality in patients with diabetes mellitus [14]. During the last two decade, main importance of chronic inflammation was highlightened with the improvements of defining the pathogenesis of atherosclerosis in this population [3]. Beyond its other detrimental effects, chronic inflammation may cause endothelial dysfunction secondary to decreasing vasodilatatory mediators including prostaglandins and nitric oxide [15]. In one hand, this chronic situation alters the vasodilatation-vasoconstriction steady state which results in the deterioration of antiatherogenic and antithrombotic properties of the endothelium. On the other hand, hypertension may become prominent secondary to diminished NO levels regardless of other factors that affects blood pressure levels in diabetic patients [16]. Inflammatory cytokines such as Tumor necrosis factor-α (TNF-α), IL-1β and IL- 6 were found to play a central role in the vicious circle of inflammation, endothelial dysfunction and atherosclerosis in patients with diabetic micro and macrovascular complications [17,18]. Furthermore, previous studies have suggested that chronic low grade inflammation might play a major role in development of insulin resistance, thus might further proceed to development of overt diabetes mellitus [15].

Recent studies demonstrated that activated neutrophils and platelets could be an important part of increased atherogenesis especially in the era of inflammation commonly seen in chronic diseases [19-21]. Platelets can interact with a variety of different cell types including endothelial cells, dendritic cells, T-lymphocytes, neutrophils and mononuclear phagocytes. Additionally, recent studies showed that the interactions of platelets with these cells mentioned above might initiate and exacerbate the inflammation in the arterial wall [22]. There has been an increasing evidence demonstrated that activated platelets could incite leukocyte recruitment to the vessel wall and trigger the inflammation that can mainly seen in the pathogenetic mechanism of atherosclerosis [23].

As novel inflammation markers, PLR and NLR were introduced in cardiac and non-cardiac disorders [5,24-30]. Moreover, NLR was shown to be related with cardiovascular morbidity and mortality in diabetic patients [31,32]. In the present study, we found that diabetic patients had higher levels of PLR and NLR than control subjects. This might be attributable to increased inflammation in this population.

Epicardial adipose tissue (EAT) originates from the splanchnopleuric mesoderm [33]. Mazurek et al. [34] concluded that, like abdominal visceral adipose tissue, EAT is also metabolically active because it can secrete proinflammatory cytokines and utilize free fatty acids (FFAs). In a recent study, the authors demonstrated that EAT acts an extremely active organ that produces several bioactive adipokines, as well as proinflammatory and proatherogenic cytokines including tumor necrosis factor (TNF)-α, interleukin (IL)-6, resistin, visfatin, omentin, leptin, plasminogen activator inhibitor-1 (PAI-1) and angiotensinogen [4,34-37]. We recently showed that EAT is increased in hemodialysis and peritoneal dialysis patients [30,38] and this active visceral fat tissue is closely related with malnutrion-inflammation-atherosclerosis/calcification syndrome (MIAC) in dialysis patients [39].

Atherogenic index of plasma, measured as the logarithmically transformed ratio of the serum triglyceride to HDL-cholesterol, may reflect the actual composition of the lipoprotein spectrum that might predict both the cardiovascular risk and effectiveness of therapy especially in cardiovascular disorders [13]. As an inexpensive research tools, anthropometric parameters are commonly used to assess the risk factors in various metabolic disorders [7]. In this regard, previous studies have shown that anthropometric parameters including waist circumference, body mass index, waist hip ratio, and waist height ratio are useful measures for predicting the incidence of type 2 diabetes mellitus in different patient groups [8,9].

In the present study, we demonstrated that AIP and EAT measurements were increased in diabetic patients when compared with control subjects. Increased EAT was found to be positively correlated with NLR, PLR, BMI, waist circumference and AIP in patients with diabetes. AIP was significantly increased in diabetic patients who have higher EAT volumes. This association might be attributed to increased levels of proinflammatory cytokines secreted by EAT. Studies have shown an important association between obesity and cardiovascular morbidity and mortality in diabetes patients [40].

Increased waist circumference, a sign of obesity, is recognized as an important risk-factor for the development of most features of metabolic syndrome, and has been linked with all-cause mortality [41-43]. There is a significant correlation between epicardial fat measured by echocardiography and waist circumference [44,45]. Additionally; the relationship between waist circumference, EAT and subclinical inflammation has been proven [34-37,46-49]. However, a subset of obese individuals who are protected from the development of metabolic disturbances has been identified and called as “metabolically healthy obese” [50]. Epicardial adipose tissue and PLR can be used as inexpensive, non-invasive and simple tests in the differentiation of metabolically healthy obese individuals and metabolically unhealthy obese patients.

In this study, we demonstrated that waist circumference and PLR were closely associated with EAT. In this regard, one potential explanation is that, risk factors of diabetes mellitus, such as obesity, smoking and physical inactivity are associated with chronic low grade inflammation. Hence, the results of the previous studies are in accord with ours and this relation might be secondary to ongoing systemic inflammation, obesity and diabetes mellitus.

Calculation of PLR and measurement of waist circumference are quite simple and cheap methods when compared other inflammatory cytokines including IL-6, IL-1β and TNF-α. Our results confirm that PLR and waist measurements might be related to increased EAT in diabetic patients. Therefore, these simple, relatively inexpensive and universally available methods can be used by internists, nephrologists and other health care staff for the first evaluation of inflammation and obesity in patients with diabetes before applying other expensive and invasive procedures.

Iacobellis et al. [51] reported the echocardiographic measurement of EAT for the first time. Measurement of EAT with echocardiography were correlated with EAT measurements by multislice computerized tomography (MSCT) as well as anthropometric and metabolic parameters [24,30]. Although many researchers advise measuring EAT using MSCT, echocardiography is a simple and inexpensive method for EAT measurement. Hence we preferred using echocardiography to measure EAT.

Our study had three main limitations. First, this was a cross-sectional analysis of diabetic patients focusing on the relationship between PLR, NLR, AIP and EAT. Second, the sample size was relatively small. Third, although EAT has a three dimensional distribution, two dimensional echocardiographic measurements may not be enough to assess the total amount. This was not a prospective controlled study, so we cannot draw cause-and-effect relationships from our findings.

## Conclusions

The relation between inflammation and adipose tissue is extremely complex in diabetic patients. However, simple calculation of PLR and measurement of waist circumference can reveal inflammation and EAT in this population. Further randomized and controlled studies evaluating the relationship between PLR, visceral and peripheral adipose tissue in diabetic patients are needed.

## Abbreviations

AIP: Atherogenic index of plasma; BMI: Body mass index; DBP: Diastolic blood pressure; EAT: Epicardial adipose tissue; HDL-C: High density lipoprotein cholesterol; IL: Interleukin; LDL-C: Low density lipoprotein cholesterol; MIAC: Malnutrion-inflammation-atherosclerosis/calcification syndrome; MSCT: Multislice computerized tomography; NLR: Neutrophils-to-lymphocyte ratio; PLR: Platelet-to-lymphocyte ratio; SBP: Systolic blood pressure; TC: Total cholesterol.

## Competing interest

The authors declare that they have no competing interest.

## Authors’ contributions

EMA: Participated in acquisition of data and analysis of data. HH: Participated in echocardiography analysis in patients and control subjects. LD: Participated in data analysis and statistical analysis. EMB: Participated in echocardiography analysis in patients and control subjects. AO: Participated in data analysis and statistical analysis. FO: Participated in data analysis and statistical analysis. UK: Participated in acquisition of data and data analysis. KT: Participated in research design, participated in the writing of the paper. Participated in data analysis. All authors read and approved the final manuscript.

## References

[B1] Coutinho M Gerstein HC Wang Y Yusuf S The relationship between glucose and incident cardiovascular events. A metaregression analysis of published data from 20 studies of 95,783 individuals followed for 12.4 years Diabetes Care 1999 22 2 233 240 10.2337/diacare.22.2.233 10333939

[B2] DeFronzo RA Ferrannini E Insulin resistance. A multifaceted syndrome responsible for NIDDM, obesity, hypertension, dyslipidemia, and atherosclerotic cardiovascular disease Diabetes Care 1991 14 3 173 194 10.2337/diacare.14.3.173 2044434

[B3] Paneni F Beckman JA Creager MA Cosentino F Diabetes and vascular disease: pathophysiology, clinical consequences, and medical therapy: part I Eur Heart J 2013 34 31 2436 2443 PubMed PMID: 23641007. Pubmed Central PMCID: 3743069. Epub 2013/05/04. eng 10.1093/eurheartj/eht149 23641007 PMC3743069

[B4] Baker AR Silva NF Quinn DW Harte AL Pagano D Bonser RS Kumar S McTernan PG Human epicardial adipose tissue expresses a pathogenic profile of adipocytokines in patients with cardiovascular disease Cardiovasc Diabetol 2006 5 1 PubMed PMID: 16412224. Pubmed Central PMCID: 1352345. Epub 2006/01/18. eng 10.1186/1475-2840-5-1 16412224 PMC1352345

[B5] Turkmen K Erdur FM Ozcicek F Ozcicek A Akbas EM Ozbicer A Demirtas L Turk S Tonbul HZ Platelet-to-lymphocyte ratio better predicts inflammation than neutrophil-to-lymphocyte ratio in end-stage renal disease patients Hemodial Int 2013 17 3 391 396 PubMed PMID: 23522328 10.1111/hdi.12040 23522328

[B6] He W Yin C Guo G Jiang C Wang F Qiu H Chen X Rong R Zhang B Xia L Initial neutrophil lymphocyte ratio is superior to platelet lymphocyte ratio as an adverse prognostic and predictive factor in metastatic colorectal cancer Med Oncol 2013 30 1 439 PubMed PMID: 23307251. Epub 2013/01/12. eng 23307251 10.1007/s12032-012-0439-x

[B7] Seidell JC Perusse L Despres JP Bouchard C Waist and hip circumferences have independent and opposite effects on cardiovascular disease risk factors: the Quebec Family Study Am J Clin Nutr 2001 74 3 315 321 PubMed PMID: 11522554. Epub 2001/08/28. eng 11522554 10.1093/ajcn/74.3.315

[B8] Stevens J Couper D Pankow J Folsom AR Duncan BB Nieto FJ Jones D Tyroler HA Sensitivity and specificity of anthropometrics for the prediction of diabetes in a biracial cohort Obes Res 2001 9 11 696 705 PubMed PMID: 11707536. Epub 2001/11/15. eng 10.1038/oby.2001.94 11707536

[B9] Sargeant LA Bennett FI Forrester TE Cooper RS Wilks RJ Predicting incident diabetes in Jamaica: the role of anthropometry Obes Res 2002 10 8 792 798 PubMed PMID: 12181388. Epub 2002/08/16. eng 10.1038/oby.2002.107 12181388

[B10] Frohlich J Dobiasova M Fractional esterification rate of cholesterol and ratio of triglycerides to HDL-cholesterol are powerful predictors of positive findings on coronary angiography Clin Chem 2003 49 11 1873 1880 PubMed PMID: 14578319 10.1373/clinchem.2003.022558 14578319

[B11] Grundy SM Cleeman JI Merz CN Brewer HB Jr Clark LT Hunninghake DB Pasternak RC Smith SC Jr Stone NJ Coordinating Committee of the National Cholesterol Education Program Implications of recent clinical trials for the National cholesterol education program adult treatment panel III guidelines J Am Coll Cardiol 2004 44 3 720 732 PubMed PMID: 15358046 10.1016/j.jacc.2004.07.001 15358046

[B12] Cockcroft DW Gault MH Prediction of creatinine clearance from serum creatinine Nephron 1976 16 1 31 41 PubMed PMID: 1244564. Epub 1976/01/01. eng 10.1159/000180580 1244564

[B13] Dobiasova M Frohlich J Sedova M Cheung MC Brown BG Cholesterol esterification and atherogenic index of plasma correlate with lipoprotein size and findings on coronary angiography J Lipid Res 2011 52 3 566 571 PubMed PMID: 21224290. Pubmed Central PMCID: 3035693 10.1194/jlr.P011668 21224290 PMC3035693

[B14] Yosefy C Hyperglycaemia and its relation to cardiovascular morbidity and mortality: has it been resolved? Acta Diabetol 2003 40 Suppl 2 S380 S388 PubMed PMID: 14704873. Epub 2004/01/06. eng 14704873 10.1007/s00592-003-0124-9

[B15] Pitsavos C Tampourlou M Panagiotakos DB Skoumas Y Chrysohoou C Nomikos T Stefanadis C Association between low-grade systemic inflammation and type 2 diabetes mellitus among men and women from the ATTICA study Rev Diabet Stud 2007 Summer; 4 2 98 104 PubMed PMID: 17823694. Pubmed Central PMCID: 2036265. Epub 2007/09/08. eng 10.1900/RDS.2007.4.98 PMC2036265 17823694

[B16] Sena CM Pereira AM Seica R Endothelial dysfunction - a major mediator of diabetic vascular disease Biochim Biophys Acta 2013 1832 12 2216 2231 PubMed PMID: 23994612. Epub 2013/09/03. Eng 10.1016/j.bbadis.2013.08.006 23994612

[B17] Rajala MW Scherer PE Minireview: the adipocyte–at the crossroads of energy homeostasis, inflammation, and atherosclerosis Endocrinology 2003 144 9 3765 3773 PubMed PMID: 12933646. Epub 2003/08/23. eng 10.1210/en.2003-0580 12933646

[B18] Nguyen DV Shaw LC Grant MB Inflammation in the pathogenesis of microvascular complications in diabetes Front Endocrinol (Lausanne) 2012 3 170 PubMed PMID: 23267348. Pubmed Central PMCID: 3527746. Epub 2012/12/26. eng 23267348 10.3389/fendo.2012.00170 PMC3527746

[B19] Koyama H Maeno T Fukumoto S Shoji T Yamane T Yokoyama H Emoto M Tahara H Inaba M Hino M Shioi A Miki T Nishizawa Y Platelet P-selectin expression is associated with atherosclerotic wall thickness in carotid artery in humans Circulation 2003 108 5 524 529 PubMed PMID: 12860908. Epub 2003/07/16. eng 10.1161/01.CIR.0000081765.88440.51 12860908

[B20] Shoji T Koyama H Fukumoto S Maeno T Yokoyama H Shinohara K Emoto M Yamane T Hino M Shioi A Nishizawa Y Platelet activation is associated with hypoadiponectinemia and carotid atherosclerosis Atherosclerosis 2006 188 1 190 195 PubMed PMID: 16313909. Epub 2005/11/30. eng 10.1016/j.atherosclerosis.2005.10.034 16313909

[B21] Imtiaz F Shafique K Mirza SS Ayoob Z Vart P Rao S Neutrophil lymphocyte ratio as a measure of systemic inflammation in prevalent chronic diseases in Asian population Int Arch Med 2012 5 1 2 PubMed PMID: 22281066. Pubmed Central PMCID: 3277482 10.1186/1755-7682-5-2 22281066 PMC3277482

[B22] Borissoff JI Spronk HM ten Cate H The hemostatic system as a modulator of atherosclerosis N Engl J Med 2011 364 18 1746 1760 PubMed PMID: 21542745. Epub 2011/05/06. eng 10.1056/NEJMra1011670 21542745

[B23] Langer HF Gawaz M Platelet-vessel wall interactions in atherosclerotic disease Thromb Haemost 2008 99 3 480 486 PubMed PMID: 18327395. Epub 2008/03/11. eng 18327395 10.1160/TH07-11-0685

[B24] Tamhane UU Aneja S Montgomery D Rogers EK Eagle KA Gurm HS Association between admission neutrophil to lymphocyte ratio and outcomes in patients with acute coronary syndrome Am J Cardiol 2008 102 6 653 657 PubMed PMID: 18773982 10.1016/j.amjcard.2008.05.006 18773982

[B25] Walsh SR Cook EJ Goulder F Justin TA Keeling NJ Neutrophil-lymphocyte ratio as a prognostic factor in colorectal cancer J Surg Oncol 2005 91 3 181 184 PubMed PMID: 16118772 10.1002/jso.20329 16118772

[B26] Dotsenko O Chaturvedi N Thom SA Wright AR Mayet J Shore A Schalkwijk C Hughes AD Platelet and leukocyte activation, atherosclerosis and inflammation in European and South Asian men J Thromb Haemost 2007 5 10 2036 2042 PubMed PMID: 17883700. Pubmed Central PMCID: 2650817. Epub 2007/09/22. eng 10.1111/j.1538-7836.2007.02711.x 17883700 PMC2650817

[B27] Turkmen K Ozcicek F Ozcicek A Akbas EM Erdur FM Tonbul HZ The relationship between neutrophil-to-lymphocyte ratio and vascular calcification in end-stage renal disease patients Hemodial Int 2013 18 1 47 53 PubMed PMID: 23819627 23819627 10.1111/hdi.12065

[B28] Turkmen K Tufan F Selcuk E Akpinar T Oflaz H Ecder T Neutrophil-to-lymphocyte ratio, insulin resistance, and endothelial dysfunction in patients with autosomal dominant polycystic kidney disease Indian J Nephrol 2013 23 1 34 40 PubMed PMID: 23580803. Pubmed Central PMCID: 3621236. Epub 2013/04/13. eng 10.4103/0971-4065.107195 23580803 PMC3621236

[B29] Turkmen K Platelet-to-lymphocyte ratio: one of the novel and valuable platelet indices in hemodialysis patients Hemodial Int 2013 17 4 670 PubMed PMID: 24015774. Epub 2013/09/11. Eng 24015774 10.1111/hdi.12095

[B30] Turkmen K Guney I Yerlikaya FH Tonbul HZ The relationship between neutrophil-to-lymphocyte ratio and inflammation in end-stage renal disease patients Ren Fail 2012 34 2 155 159 PubMed PMID: 22172001 22172001 10.3109/0886022X.2011.641514

[B31] Azab B Chainani V Shah N McGinn JT Neutrophil-lymphocyte ratio as a predictor of major adverse cardiac events among diabetic population: a 4-year follow-up study Angiology 2013 64 6 456 465 PubMed PMID: 22904109. Epub 2012/08/21. eng 10.1177/0003319712455216 22904109

[B32] Bhat T Teli S Rijal J Bhat H Raza M Khoueiry G Meghani M Akhtar M Costantino T Neutrophil to lymphocyte ratio and cardiovascular diseases: a review Expert Rev Cardiovasc Ther 2013 11 1 55 59 PubMed PMID: 23259445 10.1586/erc.12.159 23259445

[B33] Ho E Shimada Y Formation of the epicardium studied with the scanning electron microscope Dev Biol 1978 66 2 579 585 PubMed PMID: 700255. Epub 1978/10/01. eng 10.1016/0012-1606(78)90263-4 700255

[B34] Mazurek T Zhang L Zalewski A Mannion JD Diehl JT Arafat H Sarov-Blat L O'Brien S Keiper EA Johnson AG Martin J Goldstein BJ Shi Y Human epicardial adipose tissue is a source of inflammatory mediators Circulation 2003 108 20 2460 2466 PubMed PMID: 14581396 10.1161/01.CIR.0000099542.57313.C5 14581396

[B35] Kremen J Dolinkova M Krajickova J Blaha J Anderlova K Lacinova Z Haluzikova D Bosanska L Vokurka M Svacina S Haluzik M Increased subcutaneous and epicardial adipose tissue production of proinflammatory cytokines in cardiac surgery patients: possible role in postoperative insulin resistance J Clin Endocrinol Metab 2006 91 11 4620 4627 PubMed PMID: 16895955 10.1210/jc.2006-1044 16895955

[B36] Cheng KH Chu CS Lee KT Lin TH Hsieh CC Chiu CC Voon WC Sheu SH Lai WT Adipocytokines and proinflammatory mediators from abdominal and epicardial adipose tissue in patients with coronary artery disease Int J Obes 2008 32 2 268 274 PubMed PMID: 17878891 10.1038/sj.ijo.0803726 17878891

[B37] Fain JN Sacks HS Buehrer B Bahouth SW Garrett E Wolf RY Carter RA Tichansky DS Madan AK Identification of omentin mRNA in human epicardial adipose tissue: comparison to omentin in subcutaneous, internal mammary artery periadventitial and visceral abdominal depots Int J Obes 2008 32 5 810 815 PubMed PMID: 18180782 10.1038/sj.ijo.0803790 18180782

[B38] Tonbul HZ Turkmen K Kayikcioglu H Ozbek O Kayrak M Biyik Z Epicardial adipose tissue and coronary artery calcification in diabetic and nondiabetic end-stage renal disease patients Ren Fail 2011 33 8 770 775 PubMed PMID: 21770856. Epub 2011/07/21. eng 10.3109/0886022X.2011.599913 21770856

[B39] Turkmen K Kayikcioglu H Ozbek O Solak Y Kayrak M Samur C Anil M Zeki Tonbul H The relationship between epicardial adipose tissue and malnutrition, inflammation, atherosclerosis/calcification syndrome in ESRD patients Clin J Am Soc Nephrol 2011 6 8 1920 1925 PubMed PMID: 21757644. Pubmed Central PMCID: 3359546. Epub 2011/07/16. eng 10.2215/CJN.00890111 21757644 PMC3359546

[B40] Schwandt P Can we slow down the global increase of adiposity? Int J Prev Med 2011 2 3 115 116 PubMed PMID: 21811650. Pubmed Central PMCID: 3143521. Epub 2011/08/04. eng 21811650 PMC3143521

[B41] Rexrode KM Carey VJ Hennekens CH Walters EE Colditz GA Stampfer MJ Willett WC Manson JE Abdominal adiposity and coronary heart disease in women JAMA 1998 280 21 1843 1848 PubMed PMID: 9846779 10.1001/jama.280.21.1843 9846779

[B42] Rexrode KM Buring JE Manson JE Abdominal and total adiposity and risk of coronary heart disease in men Int J Obes Relat Metab Disord 2001 25 7 1047 1056 PubMed PMID: 11443505 10.1038/sj.ijo.0801615 11443505

[B43] Visscher TL Seidell JC Molarius A van der Kuip D Hofman A Witteman JC A comparison of body mass index, waist-hip ratio and waist circumference as predictors of all-cause mortality among the elderly: the Rotterdam study Int J Obes Relat Metab Disord 2001 25 11 1730 1735 PubMed PMID: 11753597 10.1038/sj.ijo.0801787 11753597

[B44] Shetty R Vivek G Naha K Nayak K Goyal A Dias LS Correlation of epicardial fat and anthropometric measurements in Asian-Indians: a community based study Avicenna J Med 2012 2 4 89 93 PubMed PMID: 23826555. Pubmed Central PMCID: 3696206 10.4103/2231-0770.110739 23826555 PMC3696206

[B45] Kim SJ Kim HS Jung JW Kim NS Noh CI Hong YM Correlation between epicardial fat thickness by echocardiography and other parameters in obese adolescents Korean Circ J 2012 42 7 471 478 PubMed PMID: 22870081. Pubmed Central PMCID: 3409396 10.4070/kcj.2012.42.7.471 22870081 PMC3409396

[B46] Saijo Y Kiyota N Kawasaki Y Miyazaki Y Kashimura J Fukuda M Kishi R Relationship between C-reactive protein and visceral adipose tissue in healthy Japanese subjects Diabetes Obes Metab 2004 6 4 249 258 PubMed PMID: 15171748 10.1111/j.1462-8902.2003.0342.x 15171748

[B47] Park HS Park JY Yu R Relationship of obesity and visceral adiposity with serum concentrations of CRP, TNF-alpha and IL-6 Diabetes Res Clin Pract 2005 69 1 29 35 PubMed PMID: 15955385 10.1016/j.diabres.2004.11.007 15955385

[B48] Lichtash CT Cui J Guo X Chen YD Hsueh WA Rotter JI Goodarzi MO Body adiposity index versus body mass index and other anthropometric traits as correlates of cardiometabolic risk factors PLoS One 2013 8 6 e65954 PubMed PMID: 23776578. Pubmed Central PMCID: 3679008 10.1371/journal.pone.0065954 23776578 PMC3679008

[B49] Engeli S Feldpausch M Gorzelniak K Hartwig F Heintze U Janke J Mohlig M Pfeiffer AF Luft FC Sharma AM Association between adiponectin and mediators of inflammation in obese women Diabetes 2003 52 4 942 947 PubMed PMID: 12663465 10.2337/diabetes.52.4.942 12663465

[B50] Plourde G Karelis AD Current issues in the identification and treatment of metabolically healthy but obese individuals Nutr Metab Cardiovasc Dis 2014 24 5 455 459 PubMed PMID: 24529490 10.1016/j.numecd.2013.12.002 24529490

[B51] Iacobellis G Willens HJ Echocardiographic epicardial fat: a review of research and clinical applications J Am Soc Echocardiogr 2009 22 12 1311 1319 quiz 417–8. PubMed PMID: 19944955 10.1016/j.echo.2009.10.013 19944955

